# Association Between Carotid Artery Intima-Media Thickness and Combinations of Mild Cognitive Impairment and Pre-Frailty in Older Adults

**DOI:** 10.3390/ijerph16162978

**Published:** 2019-08-19

**Authors:** Jinkee Park, Jong-Hwan Park, Hyuntae Park

**Affiliations:** 1 Department of Sport Rehabilitation, Dong-Ju College, Busan 49318, Korea; 2 Health Convergence Medicine Research Group, Biomedical Research Institute, Pusan National University Hospital, 179, Gudeok-Ro, Seo-Gu, Busan 49241, Korea; 3 Department of Health Care Science, Dong-A University, Busan 49315, Korea; 4 Institute of Convergence Bio-Health, Dong-A University, Busan 49201, Korea

**Keywords:** mild cognitive impairment, pre-frailty, carotid intima-media thickness, cardiovascular disease, older adults

## Abstract

Carotid intima-media thickness (CIMT) has been proposed as a surrogate marker of cardiovascular disease. Mild cognitive impairment (MCI) and pre-frailty are reportedly associated with increased CIMT. As the evidence on the association of CIMT with combinations of MCI and pre-frailty is limited, this association is examined. A total of 231 older adults participated. MCI was defined according to clinical consensus or psychometric criteria by a dementia specialist, and considering detailed neuropsychological assessments. Also, pre-frailty was defined as subjects with frail component of 1 or 2. Carotid variables were measured using a B-mode ultrasound. The analysis of covariance (ANCOVA) was performed to assess independent differences in CIMT among the four groups, according to the cognitive function and frailty status after a multivariate adjustment. Increased CIMT is associated with combinations of MCI and pre-frailty. ANCOVA showed that CIMTs were significantly different among the four groups according to the cognitive function and frailty status. CIMTmax combined with MCI and pre-frailty was the thickest (1.04 ± 0.3 mm), whereas the CIMT of no MCI and no pre-frailty was the thinnest (0.82 ± 0.2 mm). The results suggest that combinations of MCI and pre-frailty are associated with increased CIMT in older adults.

## 1. Introduction

Increased carotid artery intima-media thickness (CIMT) is known as a risk factor closely related to the early induction of cardiovascular disease (CVD) in older adults [1]. Increased CIMT is also closely associated with early-onset myocardial infarction (MI), stroke, and all-cause mortality in many older adults [1,2,3]. Therefore, prevention of CIMT increase may be important in atherosclerotic disease (AD) such as CVD, MI, and stroke, as well as all-cause mortality in older adults [1,2,3].

Mild cognitive impairment (MCI) is an important condition that should be solved in an aged society [4,5], which can lead to various conflicts among societies (such as increased average annual direct medical cost per person) and individuals (physical disability or illness) [5,6,7]. Cognitive-impaired individuals also frequently suffer from comorbid psychiatric conditions such as depression, wandering, and sleep disorder, and experience reduced quality of life [8,9,10]. In addition, several reliable studies reported that CIMT positively correlated with MCI in older adults with or without disease [11,12,13]. Furthermore, a cross-sectional study by Yue et al. demonstrated that CIMT correlated with MCI even after adjusting for covariance in participants with acute ischemic stroke [14].

Frailty can highly cause harmful consequences, such as mortality, institutionalization, falls, and hospitalization in elderly people [15]. Frailty is also associated with AD, as well as adverse health outcomes and higher risk of incident cognitive disorders in older adults or elderly people [16,17,18]. In two recent studies, pre-frailty has been independently associated with the occurrence of CVD, disability, and fracture in older adults [19,20]. Furthermore, a recent study by Chang et al. reported that pre-frailty and frailty were associated with an increase in CIMT in Taiwanese people [21].

Meanwhile, combined CVD risk factors are reported to accelerate the CIMT increase in adults or older adults [22,23]. Therefore, this study aimed to examine the complex association among CIMT, cognitive function, and pre-frailty in older adults because of its limited evidences.

## 2. Materials and Methods

### 2.1. Study Participants

This cross-sectional study was performed in three local areas (Saha-Gu, Seo-Gu, and Jung-Gu) in Busan City, South Korea. A total of 412 older adults (aged 70–88 years) submitted an application to participate after reading an advertisement for the study in community and health centers. Older adults aged 70–88 years were included. The initial exclusion criteria were age ≤70 years, currently diagnosed with CVD (angina, MI), stroke, musculoskeletal disorders, and participants who have not completed all the tests. A total of <70 years (*n* = 12) and those currently with angina and myocardial infarction (*n* = 10), stroke (*n* = 9), and musculoskeletal disorders (*n* = 18) were excluded. Those who did not complete all the tests required in this study were also excluded and considered as secondary exclusion criterion, comprising 132 participants (physical function *n* = 26, physical activity *n* = 25, blood sampling *n* = 25, missing questionnaire *n* = 33, and ultrasound *n* = 33). In this study, angina, MI, stroke, musculoskeletal disorders, hypertension, diabetes, hyperlipidemia, and kidney disease were defined as those who were diagnosed and taking medications for each disease by a doctor. A questionnaire was used to obtain information on clinical outcomes and general characteristics of study population. The institutional review board approved the study, and informed consent was obtained from all participants included in the study.

### 2.2. Cognitive Impairment and Frailty Assessment

A Korean mini-mental state examination (K-MMSE), which has been widely used in clinical evaluations and research in Korea, was used to assess cognitive function. K-MMSE consists of 23 items (orientation to time and place, attention and calculation, language, and memory) with a possible score of 30 points [24]. In our study, the cognitive impairment diagnosis [25] was based on medical evaluations through a clinical interview by a dementia specialist, and considered with the detailed neuropsychological assessments and K-MMSE scores. A modified version of the original Cardiovascular Health Study frailty index was used, which has been well-validated [15,26]. Frailty was defined based on the following components: (1) Unintentional weight loss of 4.5 kg or more during the last years; (2) exhaustion, shown by answers to the center for epidemiologic studies-depression (CES-D) scale questions “I felt that everything I did was an effort” and “I could not get going” rated “moderate amount to most of the time during the last week”; (3) low activity (<383 Kcal per week for men, <270 kcal per week in women); (4) slowness, determined by a usual walking speed of 0.8 m/s or less; and weakness of grip strength (dominant grip strength of <26.0 kg for men and <17.0 kg in women. Participants with one or two components were identified as pre-frail, and those with no symptoms as robust [15].

### 2.3. Body Composition and Physical Function

After measuring the participants’ height and weight using a body composition analyzer (Inbody 370, Biospace, Seoul, Korea), body fat mass (BFM) percentage was subsequently measured using the bio-electrical impedance analysis method. Waist circumference (WC) was measured to the nearest 0.1 cm at the level of the umbilicus using a flexible plastic tape while the participant was standing. Body mass index (BMI) was calculated using the following formula: Weight (kg)/heigt^2^ (m^2^) × 100. Two days before the body composition measurement, participants were instructed to avoid any physical activity other than routine daily activity. Systolic blood pressure (SBP) and diastolic blood pressure (DBP) were measured using a mercury sphygmomanometer (HICO, Tokyo, Japan) after a 10-min rest. The physical function test used the maximum value after measuring each twice for four items. Measurement items and methods were as follows: Grip strength was measured using isometric dynamometer (TKK-5401, Japan) for the dominant arm; time up and go (TUG) was measured based on the time needed to stand up from a standard armchair, walk 2.44 m, turn, walk back the 2.44 m, and sit down again at the fastest speed; walking speed was measured at 4-m walking time, except for 1.5 m each in acceleration and deceleration zones respectively; the 6 min walking was measured based on the distance traveled for 6 min in a 20-m straight zone, where participants were asked to walk as far as possible for 6 min; and physical activity was measured using a Fitmeter (Fit. Life, Seoul, Korea), a 3-axial accelerometer. High correlation (*r* = 0.947) and high explanatory power (R^2^ = 0.897) were observed between the signal vector magnitude of the Fitmeter and the count-based values measured by the ActiGraph (wGT3x-BT, Pensacola, FL, USA) [27].

### 2.4. Laboratory Measurements

Blood samples were obtained from the antecubital vein after an overnight fast for biochemical tests. Total cholesterol (TC) and triglycerides (TG) were analyzed using the enzymatic colorimetric assay method. High-density lipoprotein cholesterol (HDL-C) was analyzed using the homogeneous enzymatic colorimetric method. Low-density lipoprotein cholesterol (LDL-C) was determined using the Friedewald equation [28]. Fasting glucose was analyzed using the enzymatic reference method with hexokinase. Insulin was analyzed using the electrochemiluminescence immunoassay method. High-sensitivity C-reactive protein (hs-CRP) was analyzed using the immunoturbidimetric assay method. Homeostasis model assessment of insulin resistance (HOMA-IR) was calculated using fasting plasma insulin and plasma glucose as [29]:

HOMA-IR = (fasting plasma insulin [μIU/mL] × fasting plasma glucose [mg/dL] × 0.0555)/22.5

### 2.5. Carotid Ultrasonography

Prior to the ultrasound measurements, all participants rested in a supine position for 10 min in a quiet, half-darkened room. Carotid artery variables were measured using a B-mode ultrasound and a 10-MHz probe (LOGIQ 3, GE Healthcare, Wauwatosa, WI, USA). The left carotid artery was measured using an ultrasound. CIMT was measured between 1 cm and 5 cm before the carotid bulb. The maximum CIMT (CIMTmax) was measured at the site of the thickest wall; the minimum CIMT (CIMTmin) was measured at the site of the thinnest wall; and the mean CIMT (CIMTmean) is an average thickness. The carotid plaque is defined as the presence of focal wall thickening of at least 50% greater than that of the surrounding vessel wall, or as a focal region with CIMT of greater than 1.5 mm that protrudes into the lumen that is distinct from the adjacent boundary [30]. The carotid artery luminal diameter (CLD) was measured from the far wall of the distal common carotid, 2 cm proximal to the carotid bifurcation. The maximum CLD was measured based on the maximum expansion of the carotid artery luminal diameter [31]. The CIMT was defined as the distance from the lumen-intima interface to the intima-adventitia interface, and CLD was defined as the distance between the near and far wall intima-media interfaces [31]. Reproducibility was assessed by test-retest in 25 participants in this study. Pearson correlation coefficients between the test-retest were 0.87 for the mean CIMT.

### 2.6. Statistical Analysis

SPSS version 19.0 (SPSS Inc., Chicago, IL, USA) was used for the statistical analysis, and measurement results were presented as averages, standard deviation, and frequency. Shapiro-Wilk test was used to assess the normality assumption. Independent sample *t*-test was used to identify between-group differences in variables according to the two groups for cognitive function, and frailty status. Moreover, the analysis of variance (ANOVA) was performed for difference among groups of variables according to cognitive function, and frailty status (no MCI and no pre-frailty group, no MCI and pre-frailty group, MCI and no pre-frail group, and MCI and pre-frailty group). In addition, the post hoc examination was performed according to the Tukey’s method. Linear regression analysis was performed for multiple correlation of CIMT, MCI, and pre-frailty. The analysis of covariance (ANCOVA) was used to evaluate independent differences in CIMTs among the four groups according to cognitive function and frailty status, after adjusting for gender, age, alcohol drinking, smoking, hypertension, diabetes, hyperlipidemia, kidney disease, carotid artery plaque, body mass index, blood pressure, walking speed, low density lipoprotein cholesterol, hs-C-reactive protein, and homeostatic model assessment-insulin resistance. The statistical level of less than *p* < 0.05 was considered as significant.

## 3. Results

In this study, 412 elderly people participated (men 80, women 332, age range 70–88 years) in the screening test. A total of 231 data were included for analysis, excluding the 181 data (consisting of participants who met the exclusion criteria and those who have not completed all the test). The participants’ general characteristics are shown in Table 1. In this study, the prevalence of MCI and pre-frailty were 77 (33.3 %) and 129 (55.8%), respectively.

Table 2 shows between-group differences according to cognitive function and frailty status. The MCI group showed a significantly different weight, BMI, BFM percent, WC, walking distance, physical activity, K-MMSE, CIMTmax, CIMTmean, and CIMTmin compared with the no MCI group. The pre-frailty group also showed significant differences in weight, BMI, SBP, grip strength, TUG, walking speed, walking distance, physical activity, TG, LDL-C, HDL-C, K-MMSE, CIMTmax, CIMTmean, CIMTmin, CLDmax, and CLDmin as compared with the no pre-frailty group.

Table 3 shows the differences among groups of variables according to cognitive function and frailty status. In the ANOVA analysis, weight, BMI, BFM percentage, SBP, grip strength, TUG, walking speed, walking distance, physical activity, K-MMSE, CIMTmax, CIMTmean, and CIMTmin were significantly different among the groups according to cognitive function and frailty status. Moreover, CIMTs (all) in the no MCI and pre-frailty group, MCI and no pre-frailty group, and MCI and pre-frailty group were significantly different compared with no MCI and no pre-frailty group. CIMTmax in the MCI and pre-frailty group was significantly different than those in the no MCI and pre-frailty group.

**Figure 1:**
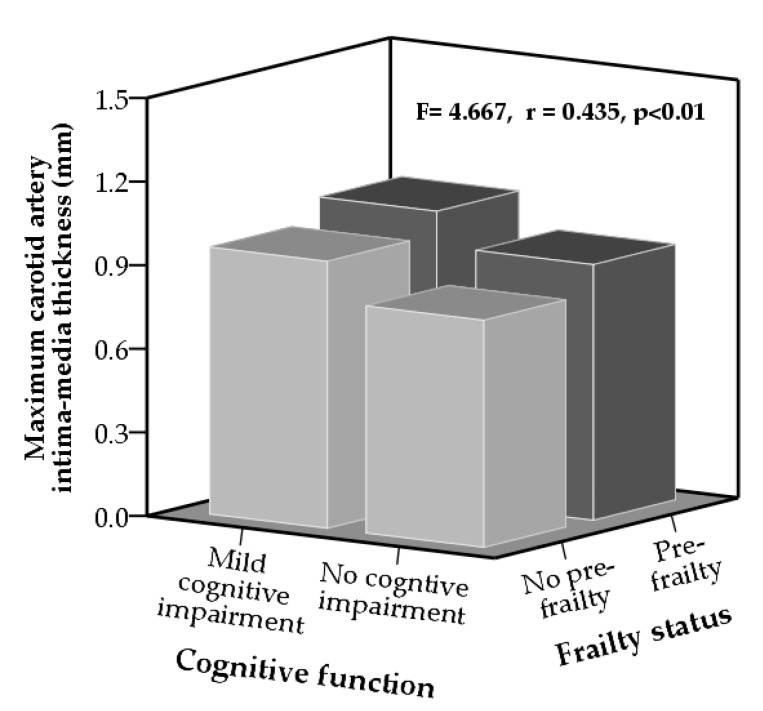
Differences in maximum carotid artery intima-media thickness according to cognitive function and frailty status after adjustment for gender, age, alcohol drinking, smoking, hypertension, diabetes, hyperlipidemia, kidney disease, carotid artery plaque, body mass index, blood pressure, walking speed, low density lipoprotein cholesterol, hs-C-reactive protein, and homeostatic model assessment-insulin resistance.

Figure 1 shows the results of multiple correlation and ANCOVA analysis. CIMTmax was significantly different among the four groups according to cognitive function and frailty status. CIMTmax was significantly correlated with cognitive function and frailty status. CIMTmax in the MCI and pre-frailty group was thickest (1.04 ± 0.3 mm), and CIMTmax in the no MCI and no pre-frailty group was thinnest (0.82 ± 0.2 mm), after adjusting for multiple variables.

F value estimated by ANCOVA analysis, and multiple correlation coefficient (*r*) was calculated by multiple lineal regression.

## 4. Discussion

This study examined the association of CIMT with cognitive function and frailty status in older adults. The primary findings are that CIMT was different among four groups according to cognitive function and frailty status. Individuals with MCI and pre-frailty had increased CIMT compared to those with no MCI and no pre-frailty; therefore, individuals with MCI and pre-frailty are more likely to have increased CIMT compared to those with no MCI and no pre-frailty.

A cross-section and follow-up study by Sander et al. reported that CIMT is thick in those with MCI than those without a baseline [32]. In addition, in their follow-up study on patients without MCI, the greater CIMT was independently associated with the progression of MCI [32]. Moreover, a recent cross-sectional study by Yue et al. reported that MCI is associated with increased CIMT in the stroke population [14]. In addition, their study showed that MCI was found to be associated with both CIMTmax and CIMTmean [14]. In our study, older adults with MCI had thick CIMTs (max, mean, and min) compared to those without MCI. Our results suggest that MCI is associated with an overall increase in the carotid artery wall thickness. In addition, our results suggest a possibility that MCI increases the risk of AD due to increased CIMT in older adults. However, because our study is a cross-sectional study, we cannot explain the causal relationship that MCI increases the risk of AD due to increased CIMT. Nevertheless, increase of carotid artery wall thickness is reported to be closely associated with increased risk of AD such as CVD, MI, and stroke [1,3,33].

Frailty is associated with AD and adverse health outcomes and higher risk of incident cognitive disorders in older or elderly participants [16,17,18]. CIMT is also closely associated with CVD and stroke in the elderly population [1,3]. Recently, a cross-sectional study on the association between CIMT and frailty status in an elderly population (mean age 64 ± 9 years) by Chang et al. demonstrated that CIMT with frailty was higher than those who were pre-frail or non frail [21]. Furthermore, their study found that CIMT with pre-frailty was also different as compared to without pre-frailty [21]. However, another cross-sectional study by Avila-Funes et al. showed that CIMT was significantly different between frailty and no pre-frailty, but no difference between pre-frailty and no pre-frailty [18]. In our study, pre-frail group had higher CIMT than those who were non-frail, with a significant difference between them. Moreover, in our study, CLD (both max and min) in the pre-frailty group was significantly wider than that in the no pre-frailty group. Therefore, our results suggest that pre-frailty in older adults is associated with negative changes in the carotid artery structure.

Two cross-sectional studies reported that combinations of CVD risk factors (aging and smoking or high BMI and WC) are associated with an accelerated increase in CIMT [34,35]. Moreover, another study by Dias et al. demonstrated that hypertensive patients with MCI have changes in the carotid vascular morphology characterized by increased CIMT [13]. Furthermore, two reliable studies recently demonstrated that combinations of MCI and frailty (or pre-frailty) are associated with increased risk of incident activities of daily living dependence and mortality, as well as increased risk of poor quality of life and incident physical limitation [36,37]. Recently, another study by Mergeani et al. reported that patients with MCI and hypertension have carotid artery changes characterized by increased CIMT [38]. In our ANOVA analysis, CIMTmax was significantly different among the four groups according to cognitive function and frailty status. Moreover, CIMTmax in the no MCI and no pre-frailty group was thinnest, whereas those in the MCI and pre-frailty group it was the thickest. In this study, CIMTmean and CIMTmin showed similar results.

In addition, results in the ANCOVA analysis, after adjusting the multivariable, showed that CIMTmax was significantly different among the groups, and CIMTmax in the MCI and pre-frailty groups was significantly (F = 4.667, *p* < 0.01) increased compared with those in the no MCI and no pre-frailty groups. Also, results in the multiple correlation by multiple lineal regression showed that CIMTmax was significantly correlated (*r* = 0.435, *p* < 0.01) with MCI and pre-frailty. Our results suggested that combinations of MCI and pre-frailty are associated with increased CIMT in older adults.

In our study, CIMTmax of MCI and pre-frailty was no different compared with MCI and no pre-frailty. The previous studies showed a higher association between MCI and CIMT than the association between frailty and CIMT in older adults [18,21,39,40]. In our study, older adults with MCI tended to have higher CIMTmax than those without MCI. This fact may be related to the absence of a difference in CIMTmax between the two groups according MCI. Therefore, prevention of MCI is important for preventing the increase of CIMT in the elderly. Despite our judgment, in our study, CIMTmax of the MCI and no pre-frailty groups was no different compared with no the MCI and pre-frailty groups. These clear relationships should be confirmed through further study.

This study has several limitations: (1) This was a cross-sectional study that examined the association of CIMT with MCI and pre-frailty. However, our results may be more inaccurate than those of follow-up or interventional studies. (2) In our study, only the left CIMT was measured. Thus, the association of CIMT with MCI and pre-frailty may be limited. However, a previous study showed that the difference in CIMT between the left and right was not significant in middle-aged and older adults [41]. (3) Our study on the association between CIMT and MCI with pre-frailty has relatively few samples (especially, men). Nevertheless, we identified a clear link between CIMT and combinations of MCI and pre-frailty in older adults.

## 5. Conclusions

This study revealed that MCI and pre-frailty were associated with increased CIMT. We also found that a combination of MCI and pre-frailty was associated with increased CIMT in older adults. Therefore, we believe that combinations of MCI and pre-frailty can accelerate the CIMT increase in the older adults.

## Figures and Tables

**Table 1:** General characteristics of study population.

Variables	Total (*n* = 231)	Cognitive Status		Frailty Status	
		No MCI (*n* = 154)	MCI (*n* = 77)	No Pre-Frailty (*n* = 102)	Pre-Frailty (*n* = 129)
Male/Female	45/186	28/126	17/60	8/94	37/92
Living alone, *n* (%)	129 (55.8)	91 (59.1)	38 (49.4)	60 (58.8)	69 (53.5)
Education					
Elementary school, *n* (%)	168 (72.7)	110 (71.4)	58 (75.3)	77 (75.5)	91 (70.5)
Middle and High school, *n* (%)	59 (25.5)	40 (26.0)	19 (24.7)	23 (22.5)	36 (27.9)
College or more, *n* (%)	4 (1.7)	4 (2.6)		2 (2.0)	2 (1.6)
Alcohol drinking					
Never, *n* (%)	73 (31.6)	49 (31.8)	24 (31.2)	41 (40.2)	32 (24.8)
Current, *n* (%)	158 (68.4)	105 (68.2)	53 (68.8)	61(59.8)	97 (75.2)
Smoking					
Never, *n* (%)	175 (75.8)	117 (76.0)	58 (75.3)	82 (80.4)	93 (72.1)
Current, *n* (%)	56 (24.3)	37 (24.0)	19 (24.7)	20 (19.6)	36 (27.9)
Comorbidities					
Hypertension, *n* (%)	74 (32.0)	55 (33.7)	19 (27.9)	32 (33.7)	42 (30.9)
Diabetes, *n* (%)	53 (22.9)	39 (23.9)	14 (20.6)	21 (22.1)	32 (23.5)
Hyperlipidemia, *n* (%)	32 (15.6)	21 (12.9)	11 (16.2)	12 (12.6)	20 (14.7)
Kidney disease, *n* (%)	18 (7.8)	13 (8.0)	5 (7.4)	8 (8.4)	10 (7.4)
Carotid artery plaque, *n* (%)	19 (8.2)	8 (4.9)	11 (16.2)	3 (3.2)	16 (11.8)

Values are frequency and percent; MCI, mild cognitive impairment.

**Table 2:** Difference between groups of variables according to the cognitive function and frailty status of subjects.

Variables	Total (*n* = 231)	Cognitive Status		Frailty Status	
		No MCI (*n* = 154)	MCI (*n* = 77)	No Pre-Frailty (*n* = 102)	Pre-Frailty (*n* = 129)
Age, years	76.8 ± 3.5	76.8 ± 3.5	76.8 ± 3.6	77.2 ± 3.7	76.5 ± 3.4
High, cm	155.6 ± 6.8	155.7 ± 6.6	155.3 ± 7.1	154.9 ± 5.9	156.1 ± 7.4
Weight, kg	60.0 ± 8.7	59.2 ± 8.6	61.6 ± 8.9 *	58.3 ± 7.8	61.3 ± 9.2 **
Body mass index, kg/m^2^	24.8 ± 3.1	24.4 ± 3.0	25.6 ± 3.2 **	24.3 ± 3.0	25.1 ± 3.1 *
Percent body fat mass, %	37.6 ± 7.7	36.8 ± 7.8	39.4 ± 7.0 *	37.9 ± 7.6	37.5 ± 7.7
Waist circumference, cm	92.6 ± 8.3	91.7 ± 8.3	94.3 ± 8.0 *	91.1 ± 8.4	93.7 ± 7.9
SBP, mmHg	136.7 ± 13.0	136.0 ± 13.2	137.9 ± 12.5	134.6 ± 12.7	138.3 ± 13.1 *
DBP, mmHg	74.3 ± 8.4	74.0 ± 8.2	74.8 ± 8.8	73.4 ± 7.7	75.0 ± 8.9
Grip strength, kg	22.1 ± 5.5	22.4 ± 5.6	21.5 ± 5.2	23.54 ± 5.4	21.0 ± 5.4 **
Time up and go, s	7.3 ± 1.4	7.3 ± 1.3	7.5 ± 1.6	7.0 ± 1.3	7.6 ± 1.5 **
Walking speed, m/s	1.22 ± 0.2	1.23 ± 0.2	1.21 ± 0.2	1.28 ± 0.2	1.18 ± 0.2 ***
Walking distance, m/6 min	375.7 ± 51.3	383.3 ± 49.9	360.4 ± 50.1 **	383.6 ± 52.9	369.4 ± 49.3 *
Physical active, kcal/week	786.1 ± 662.2	855.1 ± 732.8	648.1 ± 166.0 *	1005.9 ± 611.7	612.3 ± 651.0 ***
Total cholesterol, mg/dL	184.8 ± 30.0	183.3 ± 28.7	187.8 ± 32.3	181.0 ± 28.2	187.8 ± 31.1
Triglyceride, mg/dl	129.2 ± 49.0	127.3 ± 43.7	133.2 ± 58.4	122.1 ± 43.3	134.9 ± 52.7 *
LDL cholesterol, mg/dL	105.2 ± 28.6	103.6 ± 27.8	108.4 ± 29.6	102.9 ± 27.2	109.6 ± 29.3 *
HDL cholesterol, mg/dL	53.8 ± 12.9	54.2 ± 13.3	52.8 ± 12.0	55.7 ± 13.1	52.2 ± 12.5 *
Glucose, mg/dL	97.5 ± 14.3	97.0 ± 14.4	98.6 ± 14.1	96.4 ± 14.6	98.4 ± 14.1
Insulin, μU/mL	7.6 ± 4.5	7.4 ± 4.1	8.2 ± 5.1	7.6 ± 4.2	7.7 ± 4.7
hs-CRP, mg/L	0.61 ± 0.6	0.58 ± 0.6	0.65 ± 0.7	0.59 ± 0.6	0.62 ± 0.6
HOMA-IR	1.87 ± 1.2	1.8 ± 1.1	2.0 ± 1.4	1.8 ± 1.2	1.9 ± 1.2
K-MMSE	24.7 ± 3.2	26.5 ± 1.9	21.2 ± 2.0 ***	25.6 ± 2.7	24.1 ± 3.4 ***
CIMTmax, mm	0.92 ± 0.2	0.87 ± 0.2	1.01 ± 0.3 ***	0.85 ± 0.2	0.97 ± 0.3 ***
CIMTmean, mm	0.82 ± 0.2	0.79 ± 0.1	0.90 ± 0.2 **	0.76 ± 0.2	0.87 ± 0.2 ***
CIMTmin, mm	0.74 ± 0.2	0.71 ± 0.1	0.78 ± 0.1 ***	0.69 ± 0.1	0.77 ± 0.1 ***
CLDmax, cm	0.67 ± 0.1	0.66 ± 0.1	0.67 ± 0.1	0.65 ± 0.1	0.68 ± 0.1 *
CLDmin, cm	0.62 ± 0.1	0.62 ± 0.1	0.63 ± 0.1	0.61 ± 0.1	0.63 ± 0.1 *

Values are means ± standard deviation; MCI, mild cognitive impairment; LDL, low density lipoprotein; SBP, systolic blood pressure; DBP, diastolic blood pressure; HDL, high density lipoprotein; hs-CRP, high-sensitivity C-reactive protein; HOMA-IR, homeostatic model assessment-insulin resistance; K-MMSE, Korean version of the mini-mental state examination; CIMT, carotid artery intima-media thickness; CLD, carotid artery luminal diameter; * *p* < 0.05; ** *p* < 0.01; *** *p* < 0.001 difference between groups.

**Table 3:** Differences among groups of variables according to the cognitive function and frailty status.

Variables	No MCI and No Pre-Frailty (*n* = 79)	No MCI and Pre-Frailty (*n* = 76)	MCI and No Pre-Frailty (*n* = 24)	MCI and Pre-Frailty (*n* = 52)	ANOVA *p*-Value
Age, years	77.1 ± 3.7	76.5 ± 3.2	77.3 ± 3.4	76.6 ± 3.7	0.543
High, cm	155.2 ± 6.0	156.3 ± 7.3	153.4 ± 5.3	156.0 ± 7.7	0.273
Weight, kg	57.9 ± 7.5	60.4 ± 9.4	59.8 ± 8.8	62.6 ± 8.9 ^#^	0.023
Body mass index, kg/m^2^	24.0 ± 2.9	24.7 ± 3.1	25.4 ± 3.4	25.7 ± 3.1 ^#^	0.015
Percent body fat mass, %	37.5 ± 7.9	36.0 ± 7.7	40.0 ± 6.6	39.3 ± 7.3	0.039
Waist circumference, cm	90.9 ± 8.7	92.5 ± 7.8	93.4 ± 8.3	94.9 ± 7.9	0.056
Systolic blood pressure, mmHg	135.0 ± 13.1	137.0 ± 13.3	132.5 ± 12.0	140.5 ± 12.4	0.040
Diastolic blood pressure, mmHg	73.7 ± 7.6	74.3 ± 8.7	72.5 ± 7.8	76.0 ± 9.2	0.304
Grip strength, kg	23.4 ± 5.4	21.4 ± 5.7	23.4 ± 5.6	20.5 ± 4.8 ^#^	0.009
Time up and go, s	7.1 ± 1.2	7.4 ± 1.4	6.8 ± 1.4	7.8 ± 1.6 #	0.011
Walking speed, m/s	1.26 ± 0.2	1.19 ± 0.3	1.34 ± 0.3 ^†^	1.15 ± 0.2 ^#,‡^	0.001
Walking distance, m/6 min	387.9 ± 53.5	378.1 ± 45.5	371.1 ± 48.9	355.7 ± 52.3 #	0.005
Physical active, kcal/week	1036.7 ± 684.2	664.7 ± 733.1 ^#^	923.0 ± 366.5 ^†^	519.4 ± 458.7 ^#,‡^	<0.001
Total cholesterol, mg/dL	182.2 ± 28.5	184.0 ± 29.1	178.2 ± 26.4	192.8 ± 34.0	0.135
Triglyceride, mg/dL	124.0 ± 41.2	130.5 ± 45.9	120.7 ± 51.8	139.4 ± 61.3	0.271
LDL cholesterol, mg/dL	101.3 ± 27.8	105.7 ± 28.1	99.8 ± 25.2	112.8 ± 30.9	0.107
HDL cholesterol, mg/dL	56.1 ± 13.1	52.3 ± 13.2	54.3 ± 13.0	52.1 ± 11.6	0.213
Glucose, mg/dL	95.6 ± 13.6	98.3 ± 15.2	99.5 ± 17.1	98.4 ± 12.7	0.519
Insulin, μU/mL	7.6 ± 4.0	7.2 ± 4.2	7.9 ± 5.0	8.4 ± 5.2	0.493
hs-CRP, mg/L	0.62 ± 0.6	0.54 ± 0.6	0.49 ± 0.7	0.73 ± 0.7	0.270
HOMA-IR	1.80 ± 1.0	1.77 ± 1.2	2.06 ± 1.2	2.04 ± 1.3	0.471
K-MMSE	26.7 ± 2.0	26.3 ± 1.8	22.1 ± 1.2 ^#,†^	20.8 ± 2.2 ^#,†,‡^	<0.001
CIMTmax, mm	0.82 ± 0.2	0.92 ± 0.2 ^#^	0.96 ± 0.1 ^#^	1.04 ± 0.3 ^#,†^	<0.001
CIMTmean, mm	0.74 ± 0.2	0.84 ± 0.2 ^#^	0.86 ± 0.1 ^#^	0.92 ± 0.3 ^#^	0.003
CIMTmin, mm	0.66 ± 0.2	0.76 ± 0.2 ^#^	0.78 ± 0.2 ^#^	0.79 ± 0.2 ^#^	0.002
CLDmax, cm	0.65 ± 0.1	0.67 ± 0.1	0.66 ± 0.1	0.68 ± 0.1	0.364
CLDmin, cm	0.61 ± 0.1	0.63 ± 0.1	0.62 ± 0.1	0.64 ± 0.1	0.391

Values are means ± standard deviation; MCI, mild cognitive impairment; LDL, low density lipoprotein; HDL, high density lipoprotein; hs-CRP, high-sensitivity C-reactive protein; HOMA-IR, homeostatic model assessment-insulin resistance; K-MMSE, Korean version of the mini-mental state examination; CIMT, carotid artery intima-media thickness; CLD, carotid artery luminal diameter; ^#^ difference vs. no mild cognitive impairment and no pre-frailty group; ^†^ difference vs. no mild cognitive impairment and pre-frailty group; ^‡^ difference vs. mild cognitive impairment and no pre-frailty group.
